# Challenges and opportunities in the islet transplantation microenvironment: a comprehensive summary of inflammatory cytokine, immune cells, and vascular endothelial cells

**DOI:** 10.3389/fimmu.2023.1293762

**Published:** 2023-12-04

**Authors:** Qi-dong Chen, Long Liu, Xiao-hong Zhao, Jun-bo Liang, Shao-wei Li

**Affiliations:** ^1^Taizhou Hospital, Zhejiang University School of Medicine, Taizhou, Zhejiang, China; ^2^Department of Hepatobiliary and Pancreatic Surgery, Second Affiliated Hospital, School of Medicine, Zhejiang University, Hangzhou, Zhejiang, China; ^3^Department of Pharmacy, Taizhou Hospital, Zhejiang University , Taizhou, Zhejiang, China; ^4^Department of Gastroenterology, Taizhou Hospital of Zhejiang Province affiliated to Wenzhou Medical University, Linhai, Zhejiang, China; ^5^Key Laboratory of Minimally Invasive Techniques & Rapid Rehabilitation of Digestive System Tumor of Zhejiang Province, Taizhou Hospital Affiliated to Wenzhou Medical University, Linhai, Zhejiang, China

**Keywords:** islets transplantation, microenvironment, inflammatory cytokines, vascular endothelial cells, immune cells

## Abstract

It is now understood that islet transplantation serves as a β-cell replacement therapy for type 1 diabetes. Many factors impact the survival of transplanted islets, especially those related to the microenvironment. This review explored microenvironmental components, including vascular endothelial cells, inflammatory cytokines, and immune cells, and their profound effects on post-islet transplantation survival rates. Furthermore, it revealed therapeutic strategies aimed at targeting these elements. Current evidence suggests that vascular endothelial cells are pivotal in facilitating vascularization and nutrient supply and establishing a new microcirculation network for transplanted islets. Consequently, preserving the functionality of vascular endothelial cells emerges as a crucial strategy to enhance the survival of islet transplantation. Release of cytokines will lead to activation of immune cells and production and release of further cytokines. While immune cells hold undeniable significance in regulating immune responses, their activation can result in rejection reactions. Thus, establishing immunological tolerance within the recipient’s body is essential for sustaining graft functionality. Indeed, future research endeavors should be directed toward developing precise strategies for modulating the microenvironment to achieve higher survival rates and more sustained transplantation outcomes. While acknowledging certain limitations inherent to this review, it provides valuable insights that can guide further exploration in the field of islet transplantation. In conclusion, the microenvironment plays a paramount role in islet transplantation. Importantly, we discuss novel perspectives that could lead to broader clinical applications and improved patient outcomes in islet transplantation.

## 1 Introduction

Type I diabetes mellitus (T1DM) results from the absence of islet β cells, typically due to autoimmune attacks or surgical pancreas removal (1). The typical symptoms of high blood sugar due to insulin deficiency often manifest rapidly and include increased urination, thirst, weight loss, abdominal discomfort, and headaches. Without appropriate replacement therapy, patients may ultimately develop microvascular complications, ketoacidosis, and even death (2, 3). In 1921, Franklin Banting’s discovery of insulin revolutionized the management of T1DM, turning it into a manageable chronic condition. The development of rapid and long-acting insulins and the clinical use of insulin pumps combined with continuous glucose monitoring (CGM) have led to remarkable therapeutic advancements (4). However, the reliance on CGM, insulin pumps, dietary control, and increased physical activity places significant financial and psychological stress on patients and their families. Moreover, these exogenous therapies, CGMs and insulin pumps have a delayed detection and control of blood glucose levels, whereas pancreatic cells are able to detect blood glucose levels more quickly and accurately and deliver precisely measured amounts of insulin (5). Moreover, although intensified insulin treatment regimens can ameliorate glycated hemoglobin levels, they do not provide protection against diabetes complications (6). Pancreas transplantation becomes a consideration when patients face severe metabolic complications, incapacitating problems with exogenous insulin therapy or failure of insulin-based management to prevent acute complications (7). By transplanting an entire vascularized pancreas, we can restore the natural balance between blood glucose and insulin (8). Nevertheless, this approach remains challenging, primarily due to immunological considerations. While the matching of the donor pancreas to the recipient’s HLA type is a desirable goal to prevent hyperacute and acute rejection (9). The paramount consideration lies in ensuring compatibility, meaning the absence of pre-existing HLA antibodies specific to the donor’s HLA antigens in the recipient (10). Furthermore, the presence of postoperative complications is often a contributing factor to transplant failure (7). Hence, the concept of islet transplantation emerged as a less invasive and complication-prone cellular therapy (11). Unfortunately, many issues must be addressed to improve survival after islet transplantation, including islet viability, effective implantation, islet function, and immune response resulting in islet damage (12). Currently, islet transplantation primarily involves intrahepatic transplantation into the portal vein due to its accessibility and lower morbidity (13). Regrettably, immediate islet loss post-transplantation can be as high as 50%-70% (14). It is widely thought that transplanted islets are directly exposed to blood in the liver and its complex microenvironment, significantly contributing to this early loss. Factors include immediate blood-mediated inflammatory responses, immune reactions, and the impact of angiogenesis on the transplanted islets (15, 16).

This paper aims to summarize the influence of the microenvironment on islet survival post-transplantation, with a particular focus on inflammatory cytokines, vascular endothelial cells, immune cells, and potential strategies to address these challenges.

## 2 The process of islet transplantation

Currently, the primary source of pancreatic islets for clinical transplantation is deceased donors. An ideal donor should meet the following criteria: age between 20 and 50 years, BMI less than 30 kg/m², and HbA1c less than 6.5% (17–20).

Once the pancreas is excised, it should be promptly preserved in a cold storage solution to ensure the quality of preservation. To obtain clinically usable islet preparations, pancreatic tissue must undergo enzymatic digestion using a mixture of collagenase and protease enzymes. This process disperses acinar cells while minimizing damage to the islets.

Following the completion of enzymatic digestion, pancreatic islets must undergo purification, as impure islet preparations exhibit reduced functionality compared to their purified counterparts. Moreover, infusing larger tissue volumes from insufficiently purified islets can lead to increased portal vein pressure, raising the risk of portal vein thrombosis. Post-purification, the isolated islets can be cultured in a suitable medium for 24-72 hours to assess their functionality and viability (21).

The final pancreatic islet cell product is suspended in a transplantation culture medium and loaded into sterile infusion bags containing 70 units of heparin per kilogram of recipient body weight (20). During the surgical process, access to the portal vein system is achieved through percutaneous or minimally invasive abdominal approaches, allowing for direct infusion of the islets into the portal vein system. The islets are then retained within the small portal vein branches within the liver parenchyma, ultimately establishing microvascular blood supply (22).

## 3 Microenvironmental factors in islet transplantation

### 3.1 Vascular endothelial cells

#### 3.1.1 Relationship between vascular endothelial cell and islet survival

**Figure 1:**
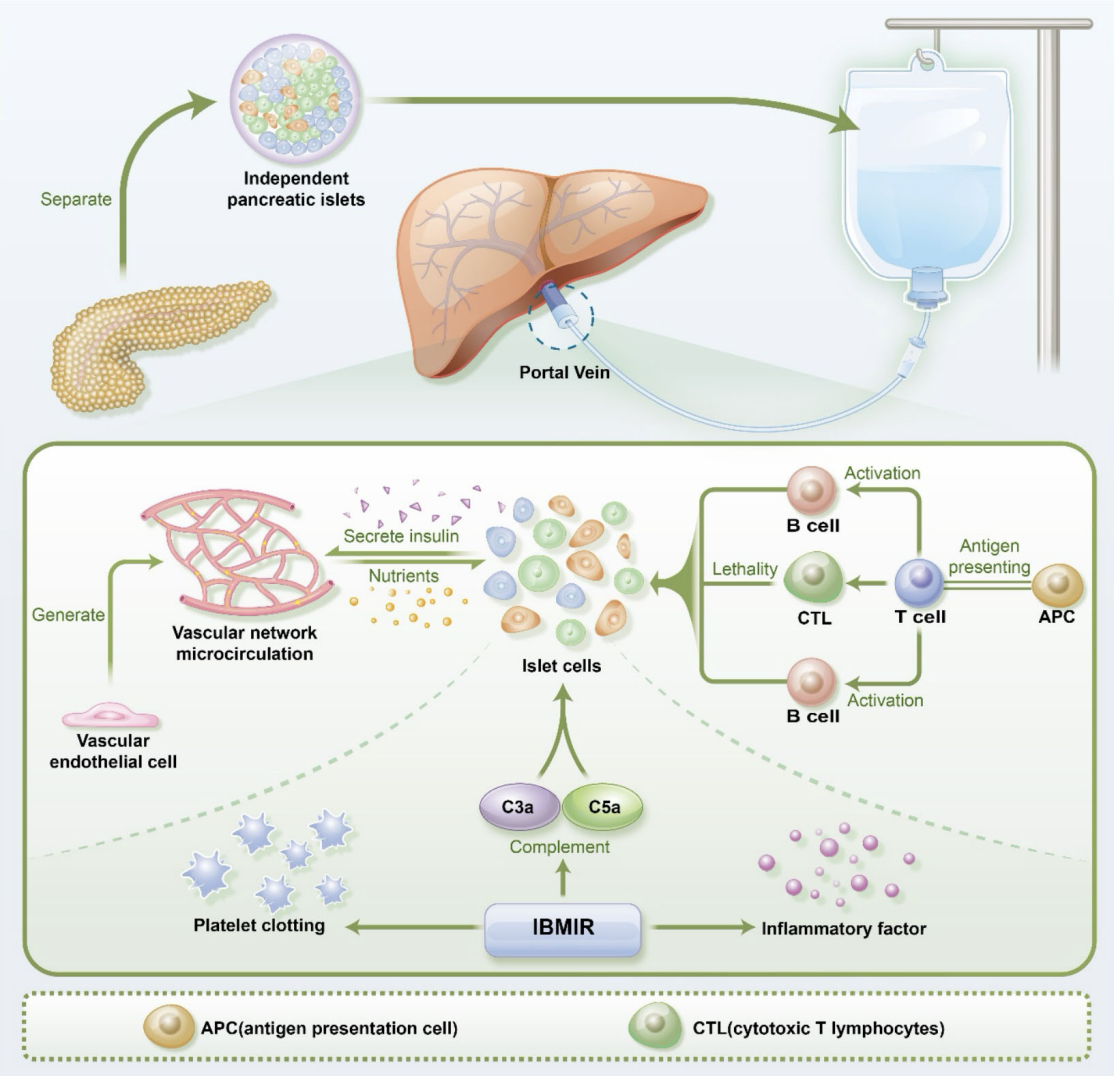
The process of pancreatic islet transplantation and the microenvironmental challenges faced by pancreatic islets, including vascular endothelial cells, immune cells, and IBMIR(immediate blood-mediated inflammatory response). APC(antigen presentation cell) CTL(cytotoxic T lymphocytes).

Endothelial cells (ECs) are a predominant cell type within the pancreatic islets, organized into a precisely regulated and morphologically distinct microcirculation network that facilitates a high degree of vascularization within the pancreatic tissue. As shown in **Figure 1**, in human islets of Langerhans (with a diameter of 40-60 µm), β-cells are located at the core, while blood vessels are situated in the periphery. In larger islets, micro vessels penetrate and branch within the islet’s interior, and the insulin produced by these β-cells is transported to the peripheral circulation through the microvasculature within the islets (23). The survival and functionality of endothelial cells are therefore paramount for rapid and efficient blood perfusion after pancreatic islet transplantation (24, 25). However, when the islets were cultured prior to transplantation, the ECs within the islets decreased rapidly and disappeared after 7 days of culture (26).

In the days following pancreatic islet transplantation, processes of angiogenesis, neovascularization, and vascular reconstruction swiftly ensue. These dynamic events primarily involve the participation of donor endothelial cells, recipient’s local vascular cells, and recruited cells from the bone marrow (27, 28). Ultimately, the vascular system formed within the transplanted islets represents a mosaic of cells from both donor and recipient origins. The reconstitution of blood flow within the transplanted islets occurs within 7-14 days, yet the post-reconstruction vascular density exhibits a reduction compared to native islets, amounting to 24% of the native pancreatic islet vascular density (29, 30).

Furthermore, an increasing body of research suggests that endothelial cells play a constructive role within the pancreatic islet microenvironment, engaging in crosstalk with β-cells (31, 32). Endothelial cells function as endocrine cells, releasing various active molecules through distinct molecular pathways, such as Hepatocyte Growth Factor (HGF), Thrombospondin-1 (TSP-1), laminin, collagen, among others, inducing nearby β-cells to differentiate, proliferate, survive, and enhance insulin secretion (33–35). Consequently, promoting post-islet transplantation vascularization may emerge as a novel therapeutic target for diabetes treatment.

Vascular endothelial growth factor (VEGF), generated by β cells within islets, plays a pivotal role in regulating islet vascular development and vascular homeostasis. In ECs, VEGF induces cell migration and proliferation and maintains fenestrations. Insufficient VEGF levels have been correlated with decreased capillary density and vascular permeability within islets, subsequently impairing their functionality (36). However, studies indicate that endogenous angiogenic factors produced by transplanted islets might be inadequate to induce angiogenesis in the early post-transplantation period (37). For instance, Montazeri et al. demonstrated that a porous collagen scaffold loaded with VEGF within rat pancreatic islet transplants facilitated vascular generation and improved graft functionality (38). Similarly, Yin et al., utilizing VEGF-conjugated alginate material to encapsulate transplanted islets, exhibited sustained angiogenic promotion upon subcutaneous transplantation (39). However, an excess of vascular endothelial growth factor (VEGF) is not universally beneficial, as its overexpression can lead to vascular dysfunction and pancreatic islet impairment (40). Therefore, investigating the appropriate VEGF concentration within the microenvironment is of paramount importance for facilitating early vascular formation in transplanted islets.

In addition to leveraging the angiogenic properties of VEGF to stimulate vascular development, cell-based adjunct therapy during transplantation represents a promising strategy for enhancing the process of pancreatic islet revascularization. Given the limited survival characteristics and plasticity of mature endothelial cells, endothelial progenitor cells (EPCs) emerge as an optimal choice. EPCs, originating from the bone marrow, possess the capacity to migrate to sites of tissue injury or ischemia and actively participate in angiogenesis and endothelial regeneration (41). Moreover, the utilization of autologous blood for EPC isolation can effectively mitigate the risk of rejection. Research conducted by Daniella et al. has demonstrated that the co-transplantation of EPCs significantly augments the engraftment rate of transplanted islets and improves initial glycemic control (42). Studies by Liza et al. further underscore that islet grafts encapsulating endothelial progenitor cells exhibit markedly enhanced blood perfusion and oxygen tension compared to control grafts (43). Beyond endothelial cells, specific cell types have also been identified with angiogenesis-promoting capabilities. Research suggests that M2-type macrophages can also stimulate neovascularization in transplanted islets, reduce cellular apoptosis, and enhance islet graft survival (44).

These findings suggest that vascular endothelial cells can improve the survival and function of transplanted islets by promoting angiogenesis.

#### 3.1.2 The effect of angiogenesis on pancreatic oxygen supply

Despite being in direct contact with the bloodstream within the portal vein, early vascularization deficiencies in transplanted islets lead to their reliance on surface oxygen diffusion rather than direct arterial perfusion with oxygenated blood (45). Research by K. E. Dionne et al. demonstrated that isolated Langerhans islets exhibited diminished insulin secretion due to hypoxia. This reduction in insulin secretion correlated with the presence of intra- and extra-islet Oxygen partial pressure gradients, resulting in a radial decline in islet cell exposure to low Oxygen partial pressure levels from the periphery to the core (46). Furthermore, under hypoxic conditions, aerobic glucose metabolism shifts to anaerobic glycolysis, ultimately triggering caspase-3 activation and islet cell apoptosis (47). Therefore, complete vascularization of transplanted islets is crucial for providing an adequate oxygen supply to reverse these phenomena.

In a study by Haofei Li et al., GelMA/HepMA/VEGF scaffolds were found to recruit human umbilical vein endothelial cells, fostering a rich vascular network around the scaffold. This augmented neovascular network significantly increased subcutaneous oxygen content, enhancing islet vitality, especially in the early stages of islet transplantation (48). Liza Grapensparr et al. enveloped human islet transplants with endothelial progenitor cells derived from umbilical cord blood and placed them in the subcapsular space of the kidney in non-obese diabetic/severe combined immunodeficiency mice. Four weeks post-transplantation, blood flow perfusion and oxygen tension of the grafts were assessed using laser Doppler flowmetry and Clark microelectrodes, respectively. Notably, islet transplants with incorporated endothelial progenitor cells exhibited significantly higher blood flow perfusion and oxygen tension compared to control grafts (43).

In conclusion, recruiting vascular endothelial cells and promoting angiogenesis are believed to ameliorate the oxygen supply situation for transplanted islets.

#### 3.1.3 The role of microcirculation on pancreatic islets

The microcirculation within pancreatic islet primarily consists of vascular endothelial cells, that facilitates the delivery of nutrients and waste clearance in response to glucose fluctuations. It achieves this while avoiding significant changes in hydrostatic pressure to preserve the integrity of islet capillary exchange (49). Traditionally, islets were considered independent of the surrounding exocrine tissue, lacking an integrated capillary network connecting the endocrine and exocrine pancreas. Blood flow within islets was believed to be unidirectional, with the sole connection between the endocrine and exocrine systems being the islet-acinar portal vein through which blood exits the islet and enters the exocrine tissue (50). Currently, there are three main models regarding the concept of islet perfusion: 1. Non-β cells being perfused before β-cells, allowing other endocrine cells to influence downstream β-cell function. 2. β-cells being perfused before other endocrine cells. In this scenario, β-cells are given a relatively high perfusion priority, so they dominate the function of the islets. 3. No distinct perfusion order. However, recent studies have indicated that both mouse and human islets are not confined to the closed “glomerulus-like” structure but rather exhibit an open arrangement where islet capillaries continuously merge with capillaries of the exocrine pancreas (51, 52). Blood flow within the islet microcirculation holds significance for islet development and the regulation of the islet hormone network, with microcirculatory abnormalities impeding insulin production and accelerating the progression of diabetes (53). In a study by Chieko Ihoriya et al. changes in islet microcirculation were investigated by administering varying doses of Angiotensin II or Angiotensin I receptor blockers via intravenous injection. Their study revealed that islet microcirculatory blood flow decreased after islet vasoconstriction, subsequently leading to reduced glucose-stimulated insulin secretion (54).

Hence, during the isolation of islets from the pancreas for transplantation purposes, the detachment not only physically separates islets from their complete capillary network but also introduces potential differences in function and structure compared to islets within the native environment. These differences may impact the functionality of transplanted islets.

In conclusion, the reconstruction of islet microcirculation is of great significance to improve the survival and reproduce the function of transplanted islets.

### 3.2 Inflammatory cytokine

#### 3.2.1 Inflammatory cytokines and islet damage

The early loss of transplanted islet vitality due to early inflammatory responses poses a significant challenge to the long-term survival rate of pancreatic islet transplantation, akin to other organ or tissue transplants. In fact, it is estimated that up to 80% of transplanted islets are lost during the initial inflammatory reaction (55).

As shown in **Figure 1**, the immediate blood-mediated inflammatory response (IBMIR) plays a key role in this process. Traditional pancreatic islet transplantation via the portal vein exposes islets directly to the blood, triggering IBMIR. IBMIR is initiated by strong activation of the coagulation cascade, where negatively charged surface of the islets activate the intrinsic coagulation pathway (56), and islet-expressed tissue factor (TF) induces the extrinsic coagulation pathway (57). This cascade activates thrombin, prompting endothelial cells to release pro-inflammatory cytokines such as interleukin-6 (IL-6) and IL-8, leading to the recruitment and accumulation of nearby neutrophils and macrophages. Simultaneously, macrophages release an array of pro-inflammatory factors, including interferon-gamma (IFN-γ), IL-1β, IL-6, and IL-8, sustaining the inflammatory response (58). The islets themselves also secrete numerous inflammatory factors like monocyte chemoattractant protein-1 (MCP-1), IFN-γ, IFN-γ-inducible protein-10 (IP-10), IL-6, and IL-8 due to hypoxia and stress (58). These pro-inflammatory factors further trigger inflammatory reactions, escalating islet cell apoptosis and causing damage to transplanted islets.

Additionally, the activation of the complement system is a crucial aspect of IBMIR, reflected in increased complement concentrations in the serum of pancreatic islet transplant recipients (59). The activation of complement proteins C3a and C5a leads to leukocyte recruitment and accumulation, upregulation of endothelial and platelet adhesion molecules, and the generation of reactive oxygen species (ROS) (60). ROS can activate the NF-κB signaling pathway through protein, lipid, and nucleic acid degradation, ultimately inducing β-cell death (61).

In summary, recipients of pancreatic islet transplants generate inflammatory responses that, influenced by various inflammatory cytokines, lead to early loss and functional deactivation of transplanted islets.

#### 3.2.2 The potential of anti-inflammatory therapy to improve islet graft survival

Considering that early inflammatory responses within the transplantation microenvironment significantly contribute to the early loss and functional decline of transplanted islets, it becomes imperative to enhance anti-inflammatory management during the peri-transplant period becomes imperative. Based on a 20-year cohort from a Canadian single-center study, the combined use of IL-1 receptor antagonist (anakinra) and TNF inhibitor (etanercept) during transplantation has shown potential to increase the likelihood of sustained graft survival (62).

Numerous preclinical studies also support the perspective of anti-inflammatory treatment to improve graft survival. Quercetin, as an inflammation-modulating compound, holds promise in ameliorating post-transplant islet injury. In an *in vitro* study, quercetin-3-o-glucoside (C3G) treatment significantly reduced inflammatory markers IL-1β and NLRP3 protein expression in grafts (63). Bilirubin is the ultimate product of heme metabolism, and numerous clinical studies have demonstrated an inverse correlation between plasma bilirubin levels and various diseases (64–67). In animal models, bilirubin has exhibited anti-inflammatory activity, including in conditions such as endotoxemia, sepsis, and ischemia-reperfusion injury (68–71). Its mechanisms of action include the inhibition of inflammatory cell infiltration and the reduction of nitric oxide (NO) production (72–74). Therefore, due to its anti-inflammatory and cellular protective properties, bilirubin is considered a potential drug for protecting transplanted islets and mitigating inflammatory damage. In research by Zhu et al., exposure of INS-1 cells, simulating rat insulinoma, to cytokine-induced inflammation (IL-1β, TNF-α, and IFN-γ) resulted in cellular damage. Bilirubin, at appropriate lower concentrations, effectively mitigated INS-1 cell viability reduction and reduced cytokine-induced cell apoptosis, thereby protecting insulin secretion functionality (75). Additionally, pre-conditioning with purified bilirubin at the isolation stage improved overall islet survival by downregulating the expression of pro-inflammatory genes (MCP-1, TNF-α) (76). Antonio Citro et al. validated in a mouse experiment that CXCR1/2 inhibitors reduced leukocyte recruitment induced by transplantation, significantly prolonging graft rejection onset in a syngeneic allograft environment (77).

Moreover, the damage response of donor islets during separation and purification activates graft inflammation, exerting negative impacts. Tissue factor (TF) is considered a “danger signal,” highly present on the islet surface, and can elicit IBMIR by activating the extrinsic coagulation pathway. Clinical outcomes of islet transplantation have been directly correlated with TF expression levels, suggesting that TF blockade represents a novel therapeutic avenue to enhance the survival rate of type 1 diabetes islet transplantation (78). Strategies to inhibit TF function have been explored, including monoclonal antibodies, inactivated FVIIa factor, small-molecule inhibitors, and siRNA (78). Another “danger signal,” high mobility group box-1 protein (HMGB1), released from donor-derived islets, often signifies adverse outcomes in transplanted islets (79). Research by Nobuhide Matsuoka et al. indicated that treatment with HMGB1-specific antibodies prevented early islet graft loss and suppressed the production of IFN-γ by NKT cells and Gr-1(+)CD11b(+) cells (80). Eun Hee Jo et al. employed the HMGB1 receptor antagonist, HMGB1 A box, as an innovative approach for the encapsulation of isolated pancreatic islets, which were subsequently co-cultured with macrophages. The findings demonstrated a notable decrease in TNF-α secretion by macrophages co-cultured with encapsulated islets compared to non-encapsulated ones. Moreover, following transplantation of the encapsulated islets into diabetic mice, there was a twofold increase in islet survival rates (81). Thus, targeting the pathways mediated by HMGB1 offers potential intervention for early islet loss.

Activation of the complement system is integral to IBMIR since it serves as a crucial mediator for the release of inflammatory cytokines. Complement-derived anaphylatoxins C3a and C5a released upon IBMIR activation are believed to participate in leukocyte recruitment and infiltration. Therefore, drugs targeting complement activation also hold potential therapeutic effects to inhibit inflammation and improve transplantation outcomes. Complement C5a receptor inhibitor peptide (C5aIP) weakens the link between complement and coagulation cascades by inhibiting the upregulation of white blood cell tissue factor expression, specifically in the liver (82). Importantly, the soluble complement receptor 1 inhibitor sCR1 and TP10 exert protective effects on post-transplant islets (83, 84).

However, drugs targeting only a fraction of IBMIR processes are unlikely to block all elements of the reaction (i.e., coagulation, complement activation, production of pro-inflammatory mediators); thus, a combination of multiple drugs is necessary to enhance post-transplant islet survival.

### 3.3 Immune cells

#### 3.3.1 Effector immune cells and regulatory immune cells

Effector immune cells refer to a specific class of cells within the immune system that play a crucial role when the body faces infection or immune challenges. These cells are primarily responsible for eliminating infectious agents or abnormal cells to maintain an effective immune response. Key effector immune cells include cytotoxic T cells (CD8+ T cells), macrophages, natural killer (NK) cells, plasma cells, and CD4+ T helper cells.

Cytotoxic CD8+ T cells play a pivotal role in graft rejection reactions. CD8+ T cells directly eliminate cells presenting non-self-antigens by releasing cytotoxic molecules, such as granules and perforins, or by inducing apoptosis through cell surface interactions, like the binding of FAS ligand (also known as CD95L) on T cells to FAS receptors on target cells (85). Activated CD8+ T cells that infiltrate transplanted organs also induce the activation of macrophages, particularly through the expression of proinflammatory cytokines, such as IFN-γ (86).

Natural killer cells (NK cells) are innate immune lymphocytes that control the spread and subsequent tissue damage caused by various types of tumors and microbial infections through MHC-independent cytotoxicity (87). Recent research indicates that NK cells also act as regulatory cells interacting with dendritic cells, macrophages, T cells, and endothelial cells, modulating immune responses accordingly (88).

Macrophages are typically characterized as proinflammatory and exhibit M1 polarization during acute rejection reactions, producing proinflammatory cytokines, which result in direct cell damage and coordination of the proinflammatory immune response (89). Their major role is phagocytosis, recognizing damaged allogeneic transplant tissue through pattern recognition receptors, such as Toll-like receptors. As antigen-presenting cells, macrophages can present alloantigens in MHC class II molecules, thereby promoting adaptive immune responses (90).

Plasma cells are another type of effector immune cell derived from B cells and form the cornerstone of humoral immunity. They enable the body to combat foreign invaders, not only by neutralizing pathogens but also by performing various effector functions, including the regulation of hypersensitivity reactions, activation of the complement cascade, and modulation of mucosal microbial communities. However, their activity can be problematic in solid organ transplantation (91). In transplantation, plasma cells can produce donor-specific antibodies (DSAs), which, by activating the complement system, lead to acute and chronic rejection, resulting in vascular damage and graft loss (92). The impact of DSAs has been extensively assessed in various solid organ transplantations (93–95).

Regulatory immune cells constitute a specialized class of cells within the immune system, primarily tasked with maintaining immune homeostasis and preventing excessive immune responses. These cells play a pivotal role in regulating immune responses, suppressing autoimmunity, and limiting inflammatory processes. They include regulatory T cells, regulatory B cells, suppressive macrophages, and NK cells.

Treg cells, a subset of CD4+ T cells, are a crucial component of regulatory immune cells. Treg cells can be categorized as thymus derived Treg cells, which develop in the thymus. Their differentiation, maintenance, and functionality are tightly regulated by the expression of the transcription factor Foxp3 (Forkhead box P3). Another pathway for Treg cell generation occurs in peripheral blood cells under the influence of antigen stimulation and the appropriate combination of cytokines, including IL-2 and transforming growth factor (TGF)-beta (96).

The interest in regulatory B cells (Bregs) dates to the 1970s, with evidence suggesting that B cells can modulate the immune system by producing “suppressive” antibodies. Regulatory B cells (Bregs) discovered in mice and humans have been shown to downregulate inflammation associated with various pathological processes, including autoimmune diseases, transplant rejection, anti-tumor responses, and infections. These cells have the capacity to produce anti-inflammatory cytokines such as IL-10, TGF-beta, and IL-35, and are considered to have the foundational capacity to induce regulatory T cells (Tregs), contributing to their regulatory potential (97).

Macrophages can exhibit both protective and pathological functions. In transplantation, macrophage activation initially occurs due to tissue damage associated with ischemia-reperfusion and may lead to early graft injury. In contrast, alternatively activated macrophages can suppress the production of proinflammatory cytokines by classically activated macrophages and facilitate wound healing and tissue repair. This repair process is highly critical in the early post-transplantation period, as wound healing helps reestablish tissue homeostasis (98).

CD4+ T helper cells play a crucial role in immune rejection. They coordinate the activation of other immune cells, such as B cells and cytotoxic T cells, to enhance the immune response against allogeneic substances. These CD4+ T cells possess the ability to produce and release various cytokines, including interferon-gamma (IFN-γ) and interleukin-2 (IL-2). Additionally, CD4+ T cells actively interact with B cells, promoting the generation of antibodies and thereby strengthening humoral immunity (99, 100).

#### 3.3.2 Relationship between immune cells and damage to transplanted islets

Like most organ transplants, immune rejection is a common occurrence in pancreatic islet transplant recipients, contributing to the loss of islet graft function (101).

Immunological reactions manifest as unexplained hyperglycemia, unexpected reduction in C-peptide levels, susceptibility events, and heightened immunological risk. It is widely acknowledged that the human immune system comprises both the innate and adaptive immune systems, featuring immune cells like macrophages, dendritic cells (DCs), natural killer (NK) cells, B cells, and T cells. Macrophages engage primarily in phagocytosis, while DCs can be categorized into lymphoid tissue-resident and non-lymphoid tissue-resident subsets, with their principal role being antigen presentation. They express major histocompatibility complex (MHC) class I and II antigens, thereby activating CD8+ cytotoxic T lymphocytes (CTL) and CD4+ helper cells. NK cells, part of the innate immune system, are known for their ability to eliminate virus-infected or cancer cells, and they can also contribute to adaptive immune responses by releasing pro-inflammatory cytokines such as IFN-γ. The cytotoxicity of NK cells is finely regulated by activating and inhibitory receptors, including human killer cell Ig-like receptors (KIRs) and mouse c-type lectin-like family receptors (102). B lymphocytes are chiefly responsible for antibody production. When the islets are transplanted into the recipient, B lymphocytes can recognize the antigens that are foreign to the organ and produce antibodies to attack these antigens, causing damage to the transplanted tissue. T lymphocytes exist in various subtypes, such as helper T cells (Th1), Th2, Th17, and regulatory T cells (Tregs). Immune rejection following transplantation initiates with the infiltration of innate immune cells, especially macrophages, into the transplanted islets, followed by donor-specific lymphocyte responses involving CD4+ and CD8+ T cells and B cells.

The activation of T cells primarily occurs through three pathways: First, DCs can directly migrate from the transplanted islets to secondary lymphoid organs, where they present donor MHC molecules, thereby activating allogeneic T cell responses. In the semi-direct pathway, DCs and other antigen-presenting cells (APCs) can phagocytose allogeneic cells, present allogeneic MHC molecules on their surface, and subsequently activate T cells. Allogeneic proteins are degraded by recipient APCs, and allogeneic peptides are presented on self-MHC molecules. These allogeneic peptide-self-MHC complexes can be recognized by T cell receptors (103).

The exogenous peptides or antigens are initially internalized and processed by antigen-presenting cells, such as dendritic cells and macrophages. These antigen-presenting cells bind antigenic fragments with major histocompatibility complex (MHC) molecules, forming MHC-antigen complexes. T cells recognize these MHC-antigen complexes through their T cell receptors (TCRs) (104). CD8+ T cells bind MHC-I-antigen complexes, while CD4+ T cells bind MHC II-antigen complexes, through their respective TCRs, subsequently activating T cells and leading to T cell proliferation and differentiation. Through the interaction of CD40-CD40L, activated T cells engage in vital crosstalk with B cells, initiating a cascade of signaling events. This interaction propels the further development of B cells, transforming them into cells with the capacity to generate antibodies, thereby strengthening the humoral immune response (105). This process holds significance in the context of organ transplantation, enhancing immune responses against allogeneic substances and potentially correlating with transplant immune rejection. In the context of transplant rejection, T cells can distinguish MHC and foreign antigens within the transplanted organ, triggering a rejection response aimed at disrupting the integrity of the transplanted organ (106, 107). Upon activation, CD8+ T cells secrete cytotoxic molecules, including perforin and granzyme B, leading to the direct killing of transplanted islet cells as presented in **Figure 1**. In contrast, CD4+ T cells do not directly harm grafts; instead, they enhance the function of CD8+ cells and secrete a range of inflammatory factors, such as TNF-α and IFN-γ, resulting in local inflammatory cell infiltration and damage to β cells in the transplanted islets (108). Furthermore, the interaction between CD4+ T cells and B cells promotes the activation of B cells, leading to their differentiation into antibody-producing cells known as Plasma B cells. These Plasma B cells produce antibodies, ultimately resulting in damage to the transplanted pancreatic islets (106).

#### 3.3.3 Potential applications for suppression of immune rejection

In conventional approaches, clinicians often employ immunosuppressive drugs (ISDs) to inhibit the proliferation and function of effector T cells, thereby attenuating the body’s rejection response (109). Early immunosuppressive regimens primarily consisted of corticosteroids, azathioprine, and cyclosporine (110). However, this therapeutic approach yielded insulin independence in only approximately 10% of patients within a year. In recent years, the development of the “Edmonton protocol” has significantly improved clinical outcomes of pancreatic islet transplantation. This novel immunosuppressive regimen involves sirolimus, low-dose tacrolimus, and induction with anti-interleukin-2 receptor antibodies. Remarkably, this regimen achieves a high rate of insulin independence, with approximately 80% of patients becoming insulin-independent within a year (111). Unfortunately, this protocol necessitates lifelong medication, which diminishes patients’ quality of life, and raises the risk of various adverse reactions, such as susceptibility to infections and potential secondary malignancies (112). In addition, because ISDs are absorbed through the intestine and islets are infiltrated directly into the bloodstream via the portal vein, ISDs would have a direct toxic effect on pancreatic islet beta cells, further reducing the survival of transplanted islets (113, 114). Consequently, the ultimate goal of pancreatic islet transplantation is to attain donor-specific immune tolerance. Indeed, there is an urgent need for new strategies to avoid lifelong use of immunosuppressive agents, enhance graft survival rates, and improve secretion function.

T cell depletion represents a promising strategy. Recent studies have shown that anti-CD3 induction therapy, by depleting a significant number of T cells, holds great potential for promoting immune suppression. An anti-CD3 immunotoxin based on diphtheria toxin has been demonstrated to induce tolerance (115). María M Coronel et al. devised an immunosuppressive regimen involving programmed death ligand-1 mediated by biomaterials to treat an allogeneic islet transplantation model. This approach was characterized by the enrichment of CD206+ programmed death 1+ macrophages and the depletion of cytotoxic T cells in the graft microenvironment (116). In addition, the induction of stable mixed chimerism by bone marrow transplantation is widely recognized as a reliable and robust method of tolerance induction (117). By mimicking central tolerance, it is possible to achieve almost complete elimination of donor-specific T cells in recipients. Selective long-term depletion of donor-specific T-cell clones in the host and donor-specific graft tolerance have been achieved in preclinical rodent models (118). However, considerations of toxicity associated with recipient preconditioning and the threat of graft-versus-host disease have hampered the clinical application of this method.

Regulatory T cells (Tregs), capable of suppressing the activation and function of effector T cells, play a crucial role in maintaining immune homeostasis (119). In recent years, the characteristics of Treg cells have been harnessed to inhibit immune rejection post-transplantation. In this regard, Dario Gerace et al. engineered stem cell-derived islet cells to secrete interleukin-10 (IL-10), transforming growth factor-β (TGF-β), and modified IL-2 in addition to targeting human leukocyte antigen (HLA) and PD-L1, recruiting Tregs to enhance immune tolerance within the graft microenvironment. Results demonstrated that engineered human islet cell grafts transplanted into non-obese diabetic (NOD) mice resisted allogeneic rejection for up to 8 weeks (120). Besides, Evelina et al. co-transplanted islets with a plasmid encoding the chemokine CCL22 into the muscle of MHC-mismatched mice, resulting in localized accumulation of Tregs due to the expression and secretion of pCCL22 in muscle cells. Consequently, the population of effector T lymphocytes around the islets decreased significantly, and the onset of immune rejection was markedly delayed compared to the control group (121). In conclusion, Ying Li et al. designed a poly(lactic-co-glycolic acid) microparticle (PLGA MP) system for the local release of TGF-β1, which, when co-incubated with CD4+ T cells *in vitro*, efficiently generated antigen-specific induced Tregs (iTregs) with potent immunosuppressive functions, providing substantial protection for the graft (122).

While Treg cell therapy continues to evolve, it indiscriminately suppresses the immune system without achieving a permanent resolution of certain diseases. Transgenic Tregs offer significant promise in addressing these issues. CAR-Treg cells, an emerging immunotherapy, employ CAR (Chimeric Antigen Receptor) technology, a synthetic receptor that empowers immune cells to selectively recognize and target specific antigens. This allows regulatory T cells to modulate immune responses and reduce inflammation to prevent the immune response from damaging the graft (123). Boardman et al. discovered that, in a human skin xenograft transplant model using immunodeficient mice, adoptively transferred CAR-Tregs were more effective in alleviating allogeneic immune-mediated skin damage caused by peripheral blood mononuclear cell transplants compared to polyclonal Tregs. *In vitro* experiments demonstrated that CAR-Tregs produced anti-inflammatory interleukin-10 (IL-10) in the presence of alloantigen (124). These findings highlight the potential benefits of CAR-Tregs in graft-specific immunosuppression. The therapeutic potential of antigen specific Tregs has been confirmed in numerous autoimmune diseases, including T1D, colitis, transplant rejection, and hemophilia (125–128).

Graft modification prior to transplantation is an excellent strategy to reduce rejection and improve clinical applicability. Ali Zafar et al. maintained isolated porcine pancreatic islet cells in a three-dimensional rotating cell culture system and allowed them to aggregate with human amniotic epithelial cells. In a porcine-mouse islet transplantation model, the stem cell-modified islets had better insulin secretion than natural islets, and the allogeneic response to them by CD4+ T cells was significantly reduced (129). This provides a new way of thinking about xenogeneic islet transplantation.

In addition to immune response inhibition, some researchers have employed islet encapsulation methods to physically isolate cells from the host using a barrier that restricts the infiltration of immune cells and antibodies while allowing the penetration of oxygen, nutrients, and insulin. Interestingly, Yesl Jun et al. prepared collagen-alginate composite fiber-encapsulated islets using a microfluidic platform to simulate the natural islet microenvironment. The results demonstrated that composite fiber-encapsulated islets exhibited higher viability and more stable insulin secretion compared to free islets (130). Su et al. designed a hydrogel network and presented inhibitory peptides against the IL-1 receptor on the surface of pancreatic islet cells and showed that these peptide-modified hydrogels were effective in protecting the encapsulated cells from specific T-lymphocyte attack (131). These results suggest that encapsulating cells and tissues in hydrogels with anti-inflammatory or immunosuppressive agents may be a novel strategy to improve the function of cells and tissues in transplantation and tissue engineering.

Due to individual variations, specific treatment regimens may not be universally applicable. In the management of transplant patients, the use of biomarkers contributes to achieving genuinely personalized therapy. Immunological biomarkers offer a better reflection of the activity of drugs (or drug combinations), going beyond mere concentration measurements and providing greater value compared to pharmacokinetic assessments for immunosuppressive agents (132). Brunet et al. conducted a comprehensive review of the application of biomarkers in transplantation, discussing three categories of biomarkers: [1] those related to rejection risk (allograft reactivity/tolerance), [2] those reflecting individual responses to immunosuppressive agents, and [3] those associated with graft dysfunction (133). The objective of individualized immunosuppression is to minimize the toxicity associated with immunosuppressive regimens, with the potential to enhance long-term allograft survival without compromising short-term allograft survival (134). Thus, optimizing immunosuppression holds significant importance in improving the clinical prognosis of pancreatic islet transplant recipients. However, current research on biomarkers remains in its preliminary stages, with numerous limitations. The immune system exhibits significant variability among different individuals, posing a challenge in the quest for universal biomarkers applicable to all patients. The immune status is a dynamic and multifaceted process influenced by various factors. Variability in biomarkers over time and in different environments may hinder accurate predictions of immune states in certain circumstances. Furthermore, the mechanisms underlying transplant immune rejection are intricate, involving multiple cell types and signaling pathways. Thus, relying on a single or limited set of biomarkers may inadequately capture the comprehensive assessment of immune status (135–137).

#### 3.3.4 Immune checkpoint blockade

Antibody-mediated immune checkpoint blockade represents a revolutionary cancer immunotherapy. These same mechanisms can be reutilized to control destructive allogeneic immune responses in the transplant setting. Currently, one of the most effective and durable immunotherapies in clinical use revolves around the programmed cell death-1 (PD-1) pathway. The PD-1/PD-L1 axis plays a pivotal role in regulating alloimmune responses in the transplant environment (138). Experimental models of fully mismatched allogeneic heart transplants have demonstrated the necessity of intact PD-1/PD-L1 interactions and blocking PD-1 results in prolonged rejection times (139). Overexpression of the immune checkpoint protein programmed death-ligand 1 (PD-L1) protects human islet-like organ allografts, enabling them to maintain glucose homeostasis for 50 days in immune-competent diabetic mice (140). Shirwan and colleagues have engineered a synthetic biomaterial platform for local delivery of a chimeric streptavidin-affibody/programmed cell death-1 ligand 1 (SA-PD-L1) protein to reprogram local immune responses to transplanted islets. In a mouse model of diabetes, only when mice received SA-PD-L1-presenting biomaterial and brief rapamycin treatment could local induction of allograft acceptance be achieved. Immunological profiling showed an increase in regulatory T cells and anergic cells following SA-PD-L1 hydrogel delivery (138).

The CD47/SIRPα pathway is involved in regulating innate and adaptive immune responses. This system negatively regulates macrophage activation and phagocytosis, adhesion, platelet activation, and antibody-dependent cell-mediated cytotoxicity and phagocytosis (141–143). It has been reported that the interaction between CD47 expressed on dendritic cells (DCs) and antibodies or SIRPα expressed on T cells can inhibit DC activation and their secretion of pro-inflammatory cytokines, leading to a weakened T cell response (144, 145). Shirwan and colleagues have constructed a chimeric structure, SA-CD47, containing the extracellular domain of CD47 modified to include a streptavidin (SA) moiety. In a murine marginal mass islet transplant model, SA-CD47-engineered islets demonstrated superior engraftment and function compared to the SA control group (146).

CTLA-4, cytotoxic T-lymphocyte-associated antigen 4, is a critical immune checkpoint protein and a negative regulator. CTLA-4 exerts its inhibitory effects by interacting with B7 molecules on antigen-presenting cells, thereby suppressing T cell activation (147). Zhang and colleagues employed inkjet-based bioprinting technology to precisely deliver trace amounts of murine CTLA4/Fc fusion protein into human decellularized dermal matrix scaffolds. These scaffolds were co-transplanted with allogeneic islets under the renal capsule, establishing an immune-regulatory microenvironment around the allogeneic islets, achieving long-term engraftment of low-dose allogeneic islet cells (148).

Fas (CD95) and Fas ligand (FasL) play significant roles in immune function, including inducing cell apoptosis and regulating T cell activation (149). Fas deficiency in mice results in abnormal accumulation of antigen-specific T cells during chronic viral infections and under steady-state conditions (150, 151). Furthermore, loss-of-function mutations in genes encoding Fas and FasL lead to autoimmune lymphoproliferative syndrome (ALPS), suggesting the role of Fas and FasL in controlling lymphocyte proliferation and maintaining immune tolerance (152). Michael Skoumal and colleagues modified allogeneic islets with biotin and transiently displayed SA-FasL on their surface in a peritoneal fat pad using a micro-porous scaffold. After a short course (15 days) of rapamycin treatment, they observed sustained survival (153).

## 4 Non- hepatic transplant site

The portal vein/liver is currently the preferred site for clinical islet transplantation, accounting for 90% of clinical islet transplantations. However, early extensive islet damage due to the influence of the portal vein microenvironment has been observed following transplantation. The development of alternative transplantation sites may make it possible to implement strategies to modulate the islet microenvironment in ways not currently feasible in the liver, thereby improving survival and transplantation outcomes (154, 155).

Benjamin and colleagues suggest further research into the subcapsular space below the kidney as a site for clinical islet transplantation. This anatomical location may avoid early IBMIR-mediated damage to the islets and may promote vascular reconstruction (156).

Intramuscular and subcutaneous spaces are important candidates, as the transplantation and biopsy procedures are simple, minimally invasive, and have fewer complications. Although these sites are characterized by low vascularity and hypoxia, many experimental trials have been conducted to enhance outcomes of intramuscular and subcutaneous islet transplantation, with a focus on early vascularization of the transplanted islets (157).

Lonnie D. Shea and colleagues report the use of a proteolytically degradable synthetic hydrogel functionalized with vasculogenic factors for localized delivery, engineered to deliver islet grafts to extrahepatic transplant sites through *in situ* gelation under physiological conditions. These hydrogels induced differences in vascularization and innate immune responses among subcutaneous, small bowel mesentery, and epididymal fat pad transplant sites, with improved vascularization and reduced inflammation observed at the epididymal fat pad site. This biomaterial-based strategy improved the survival, engraftment, and function of individual pancreatic islet grafts (158).

The spleen has been studied as a candidate site for islet transplantation for a long time. Its advantages include physiological insulin drainage and immune regulation, which have recently been demonstrated to contribute to islet regeneration. Additionally, the spleen serves as a reservoir for mesenchymal stem cells that aid in tissue repair (159).

Zhen Liang and colleagues successfully implanted human pluripotent stem cell-derived islets into the abdominal transplantation site - the rectus sheath of eight non-human primates (5 males and 3 females), improving blood glucose control in diabetic primates (160).

These results suggest that non-hepatic sites as transplantation targets are worthy of further exploration.

## 5 Conclusion

In this comprehensive review, we have meticulously summarized the impact of the microenvironment on pancreatic islet transplant survival. We emphasize the pivotal role of inflammatory cytokines, vascular endothelial cells, and immune cells in enhancing overall transplant outcomes.

Preserving the functionality of vascular endothelial cells is the cornerstone for improving transplant survival. Controlling the levels of inflammatory factors helps to reduce the damage of the graft caused by the early inflammatory response; however, further research is needed to explore how to maintain defense against pathogenic microorganisms while suppressing undesired immune response against the graft to ensure the safe survival of the transplants. In the realm of immune cells, achieving a delicate balance is of paramount importance. Efforts are being made toward advances in individualized immunosuppression, immune modulation therapies, cell engineering, novel drug formulations, and immune checkpoint blockade for more precise immune regulation and suppression. Additionally, non-hepatic transplant sites also warrant further exploration.

In conclusion, the microenvironment profoundly influences the success of pancreatic islet transplantation. Future research should prioritize the fine-tuning of the microenvironment to enhance transplant efficacy.

## Author contributions

Q-DC: Writing – original draft. LL: Writing – original draft. X-HZ: Writing – original draft. J-BL: Writing – review & editing. S-WL: Writing – review & editing.
